# Fine Mapping and Screening of Candidate Gene for Yellow-to-Green Mutation in Snap Bean (*Phaseolus vulgaris* L.) Leaf Color

**DOI:** 10.3390/ijms26115293

**Published:** 2025-05-30

**Authors:** Chang Liu, Dajun Liu, Guojun Feng, Taifeng Zhang, Xiangkai Qin, Zhuang Sun, Zhishan Yan, Xiaoxu Yang

**Affiliations:** Horticulture Department, College of Advanced Agriculture and Ecological Environment, Heilongjiang University, 74 Xuefu Road, Harbin 150080, China; liuchang3126@hotmail.com (C.L.); 2016003@hlju.edu.cn (D.L.); feng998@126.com (G.F.); 2024056@hlju.edu.cn (T.Z.); 2017008@hlju.edu.cn (X.Q.); 2017028@hlju.edu.cn (Z.S.)

**Keywords:** snap bean, leaf color, mutant, fine mapping, *ytg-1*

## Abstract

The yellow-to-green mutation in plant leaf color can be easily identified and used as a marker trait for seed purity identification, improved variety breeding, hybrid purity identification, and hybrid production. In this study, the leaves of yellow-to-green mutant *ytg-1* of snap bean were selected as the experimental material, and the physiological mechanism underlying the leaf color change was studied. The mechanism was observed to belong to the lack of recovery type in the total chlorophyll stage. The decrease in chlorophyll content was due to the inhibition of the synthesis of Proto IX to Mg Proto IX. Genetic analysis revealed that a single recessive gene, *pvytg-1*, controlled the mutation trait. The gene was located in the 80-kb region of chromosome 10. Overall, six genes were observed within this interval, and based on gene functional annotation, *Phvul.010G041700* was identified as the candidate gene. Sequencing and identification of *Phvul.010G041700* revealed a single base insertion in *ytg-1* compared with the wild-type, resulting in premature termination of the gene. The results of this study will facilitate the breeding and genetic improvement of snap bean in the future.

## 1. Introduction

Leaf color is an important phenotypic feature of plants. Various types of leaf color exist in nature, providing different functional requirements for plants. Normal leaves have a green phenotype because their chlorophyll content is higher than carotenoid content. When the content or proportion of pigments in leaves changes, the leaf color may exhibit some variation. Leaf color variation is mainly manifested by the mutation in green color, which results in white coloration, yellow coloration, stripes, etc. It is usually accompanied by changes in physiological and biochemical processes and molecular level regulation, e.g., changes in carbon assimilation ability, plastid development, assembly and operation of photosynthesis system, and pigment metabolism. Research on mutations in leaf color has been conducted since the 1930s [1]. However, for a long time, attention has not been paid to it because it often leads to crop yield reduction or even death. Leaf color mutation has been studied in rice [2], wheat [3], corn [4] and other crops [5]. However, it was later discovered that leaf color mutants can be used to study chlorophyll synthesis. The yellow-to-green mutation in leaves refers to a type of leaf color mutation in which the yellow leaf color gradually reverts to green with the progression of growth and development of etiolated plants. Because the yellow-to-green leaves indicate less physiological damage to plants and are more ideal than other leaf color markers, they are of great significance for seed production and rapid identification of purity of variety.

Chlorophyll content in leaves significantly affects the leaf color phenotype of plants. The metabolic pathway of chlorophyll is a complex process co-catalyzed by various enzyme complexes. The chlorophyll reaction in plants includes four steps: the formation of 5-aminolevulinic acid (ALA), protoporphyrin IX (PPIX), and chlorophyll (involving the chlorophyll cycle), and the degradation of chlorophyll. The disruption of any reaction step will result in abnormal chlorophyll synthesis, leading to leaf color variation. Mutation in the genes responsible for chlorophyll synthesis is the direct cause of abnormal chlorophyll synthesis in plants. Zhang et al. reported that, when *Chl1* and *Chl9* encoding two subunits of rice magnesium chelatase, CHLD and CHLI, respectively, were mutated, the mutant leaves exhibited white or yellow–green phenotype [6]. Zhu et al. reported that the downregulation of *HEMC*, *HEME*, and *PORA* in the chlorophyll synthesis pathway of rice mutant ‘*ygl8*’ led to the yellow–green phenotype in its leaves [7].

At present, numerous relevant studies have confirmed that the chloroplast structure in the mutants with leaf color variation is significantly different from that in plants with normal leaves, and three mechanisms are reported that cause the abnormal development of plant chloroplasts [8,9]. The first is abnormal chloroplast differentiation and development. Any gene mutation in this process will lead to the abnormal differentiation and development of plant chloroplasts, thus affecting the chlorophyll content and ultimately leading to the variation in leaf color. For example, in the rice albino mutant ‘*al2*’, *AL2* is involved in the splicing of chloroplast group I introns [10]. The mutation of this gene leads to the destruction of chloroplast structure at an early stage, resulting in leaf albinism. The second mechanism is abnormal chloroplast function caused by the blocked synthesis of chloroplast proteins. For example, in darkness, the expression of the *Arabidopsis* plastid gene ‘*thf1*’ is blocked, resulting in a mutant with light blunt leaf yellowing. Moreover, the genes related to chlorophyll biosynthesis are synchronously inhibited, indicating that abnormal plastid development is the main reason for the leaf color variation [11]. The third mechanism is the deletion and rearrangement of plastid genes. The barley mutant albostrians exhibits the absence of plastid ribosome, which leads to the loss of nuclear coding plastid protein, abnormal chloroplast development, and poor chlorophyll synthesis, resulting in plant albinism. In addition, blocked chloroplast protein transport, gene variation in the phytochrome metabolism pathway, and gene variation in the nuclear cytoplasmic signal transduction pathway will lead to leaf color variation.

The mutation in leaf color can usually occur at the seedling stage; therefore, it is widely used in the cross breeding and fine variety breeding of crops. Using leaf color mutation as a marker character in breeding can remove false hybrids at the seedling stage, simplify the process of purity identification and improving breeding efficiency. It has been used as a marker character to distinguish false hybrid plants in hybrid rice, cotton, and other crops in China. During the process of constructing a mutation library using snap bean variety A18, many mutants with leaf color variation were obtained by us. Among them, the snap bean leaf yellow-to-green mutant *ytg-1* is a leaf color mutant that can be stably inherited. In this study, we analyzed the physiological mechanism of leaf color variation in mutant *ytg-1* by assessing its agronomic traits, photosynthetic pigment content, and the intermediate metabolites of chlorophyll. Genetic analysis, gene mapping, and transcriptome analysis were conducted for the mutant traits, and candidate genes were located and cloned. This study would provide insights into the genetic improvement and hybrid breeding of snap beans in the future.

## 2. Results

### 2.1. Morphological Characteristics of ytg-1 Mutant

**Figure 1:**
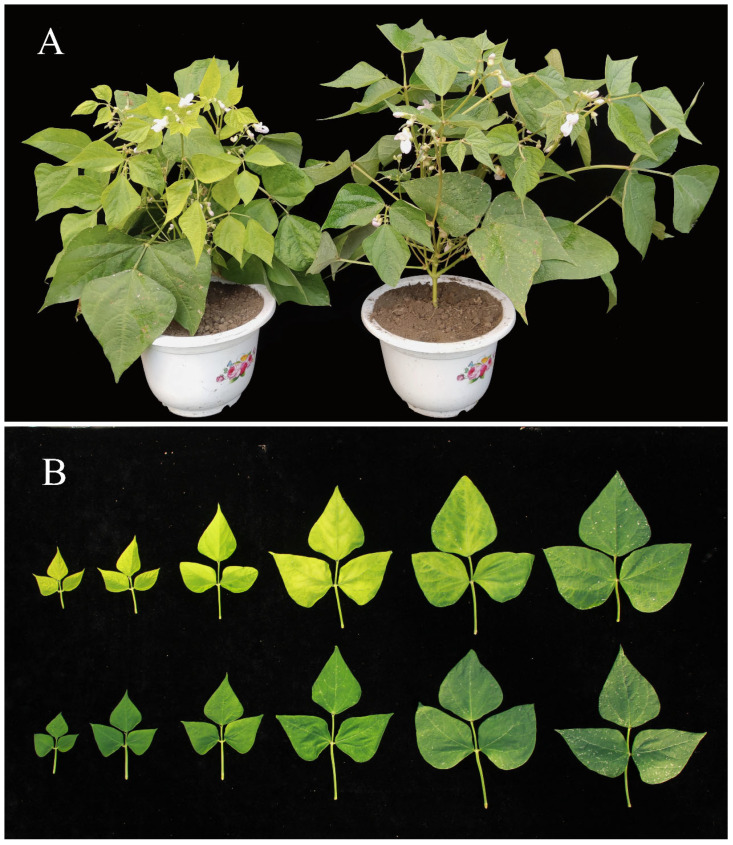
Comparison of the phenotypes of snap bean wild-type A18 and mutant *ytg-1*. Note: (**A**) Mutant *ytg-1* on the left, wild-type A18 on the right; (**B**) Comparison of leaf color between *ytg-1* and A18 during the growth period of 6–21 days, with the first row being *ytg-1* and the second row being A18.

Compared with the wild-type A18, the overall growth potential of the *ytg-1* mutant was weak (Figure 1A). Both the cotyledons and primary true leaves of *ytg-1* were green, exhibiting yellow leaves starting from the first three-compound-leaf stage. As the plant grows and develops, it gradually returns to the green leaf phenotype. Each newly grown three-compound leaf undergoes a growth process of first yellowing and then turning green, which takes approximately 21 days (Figure 1B). A18 exhibited green leaves throughout the entire developmental stage.

### 2.2. Changes in Agronomic Traits of ytg-1 Mutant

**Figure 2:**
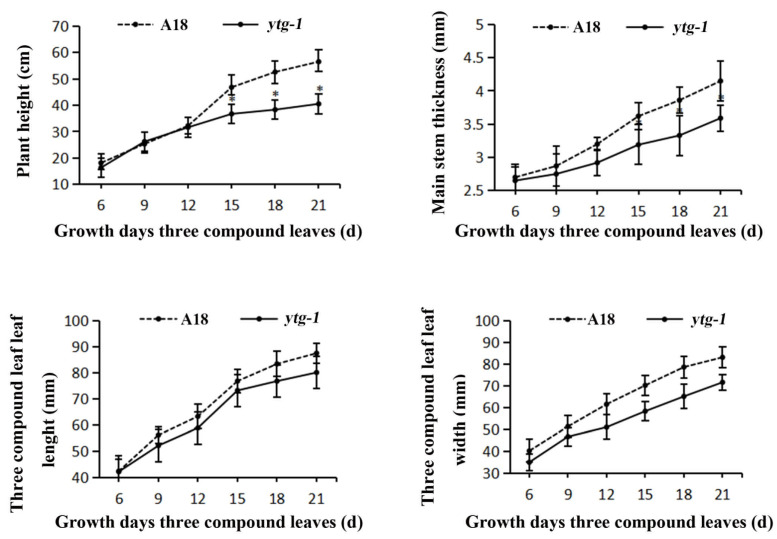
Comparison of the growth curves of mutants *ytg-1* and A18 during the growth period of 6–21 days. Note: *t* test, *: *p* < 0.05.

To further investigate the impact of the yellow-to-green leaf color trait on plant growth and development, six time points were selected to measure the plant height, main stem thickness, and length and width of three-compound leaves of A18 and *ytg-1* plants (Figure 2). The results revealed that *ytg-1* exhibited lower plant height, main stem thickness, and length and width of three-compound leaves than wild-type A18. Significant differences were observed in terms of plant height, but not in terms of other parameters. The overall growth state of *ytg-1* was weaker than that of A18. The measured yield traits are shown in Table 1. *ytg-1* exhibited significantly lower pod length compared with A18. The yield per plant of *ytg-1* was 11.1% higher than that of A18. The number of pods per plant of *ytg-1* significantly increased by 18.1% compared with A18, and the 100-grain weight significantly increased by 3.4%. The above data indicated that the yield of mutant *ytg-1* was not significantly different from A18, indicating that this yellow-to-green leaf color mutation had little impact on plant yield.

### 2.3. Analysis of Photosynthetic Pigment Content

**Figure 3:**
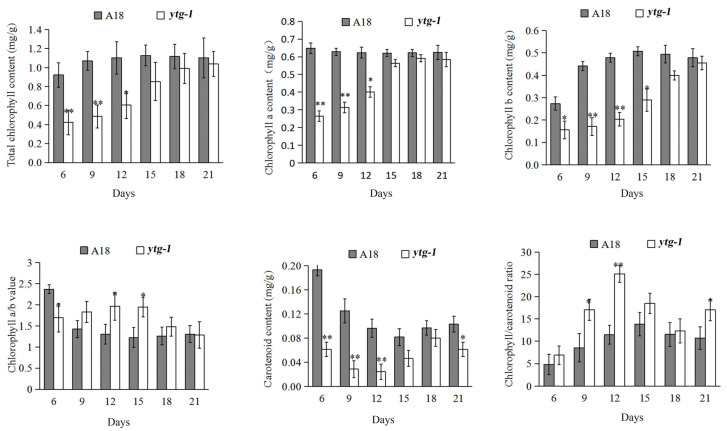
Comparison of photosynthetic pigment content in the three-compound leaves of mutant *ytg-1* and A18 during 6–21 days. Note: *t* test, *: *p* < 0.05, **: *p* < 0.01.

Photosynthetic pigments from the three-compound leaves of *ytg-1* and A18 were extracted during the growth period of 6–21 days. Before 12 days, the Chl a and b, total chlorophyll, and carotenoid contents in *ytg-1* were significantly lower than those in A18 (Figure 3). As the leaves turn from yellow to green, the contents of Chl a and b, total chlorophyll, and carotenoids in *ytg-1* exhibited an upward trend during the 12–18 days of growth period (Figure 3). During the 18–21 days of growth period, the contents of Chl a and carotenoids in *ytg-1* slightly decreased, whereas those of Chl b and total chlorophyll still exhibited an upward trend (Figure 3). Therefore, we speculated that the leaf phenotype of early yellowing and later turning green in *ytg-1* is due to a periodic lack of total chlorophyll content.

### 2.4. Analysis of the Content of Intermediate Metabolites in Chlorophyll Synthesis in ytg-1

**Figure 4:**
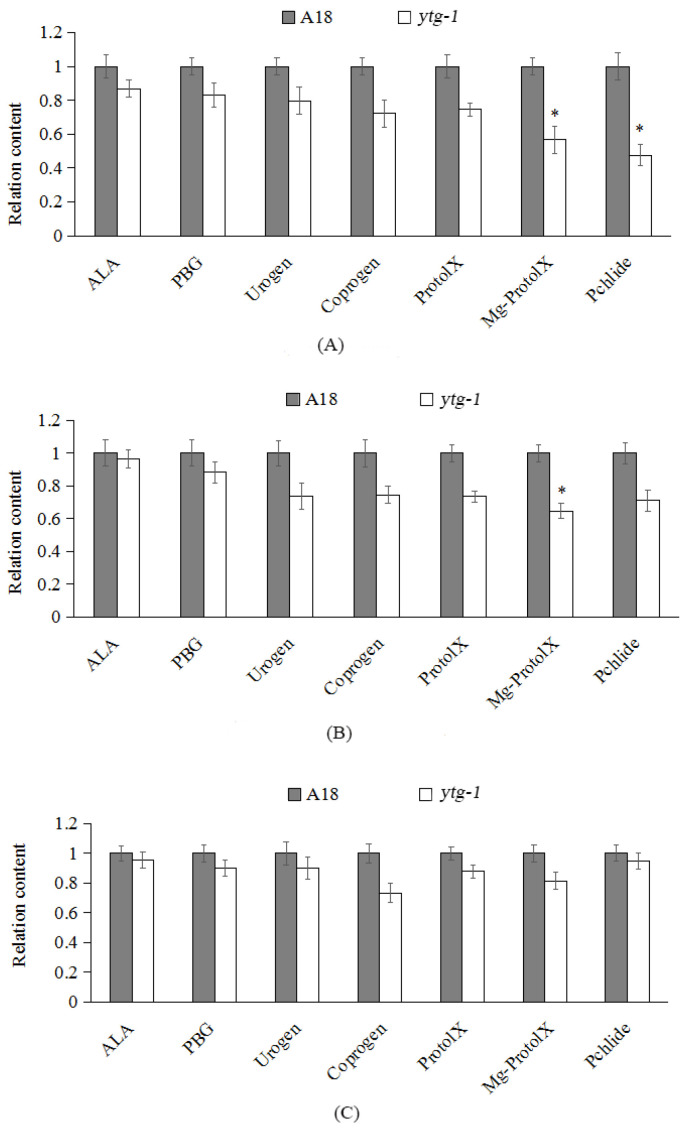
Comparison of the content of chlorophyll during yellow leaf, green turning, and late green periods in *ytg-1* and A18. Note: (**A**) yellow leaf period, (**B**) green turning period, and (**C**) late green period. *t* test, *: *p* < 0.05.

To investigate whether or not the phenotype of the yellow-to-green mutant *ytg-1* is caused by the obstruction of an intermediate metabolite of chlorophyll synthesis process, the content of intermediate metabolites in chlorophyll synthesis were measured at the yellow leaf period (6 days), green turning period (15 days), and late green period (21 days) (Figure 4). The results revealed that, during the yellow leaf period, the contents of ALA, PBG, Uogen, Coprogen, and ProtoIX in *ytg-1* leaves were lower than those in A18; however, the difference was not significant. The contents of Mg-Proto IX and Pchlide in *ytg-1* were significantly different from those in A18. During the green turning period, compared with A18, except for the Mg-Proto IX content, no significant difference was observed in the content of other intermediate metabolites. In the late green period, the relative content of intermediate metabolites in *ytg-1* was lower than that in A18; however, the difference was not significant. Therefore, it was inferred that the yellow leaf phenotype of *ytg-1* may be due to the hindered steps of synthesis from Proto IX to Mg Proto IX, which in turn affected the synthesis of Chl a, Chl b, and total chlorophyll.

### 2.5. Ultrastructural Analysis of Chloroplast in ytg-1

**Figure 5:**
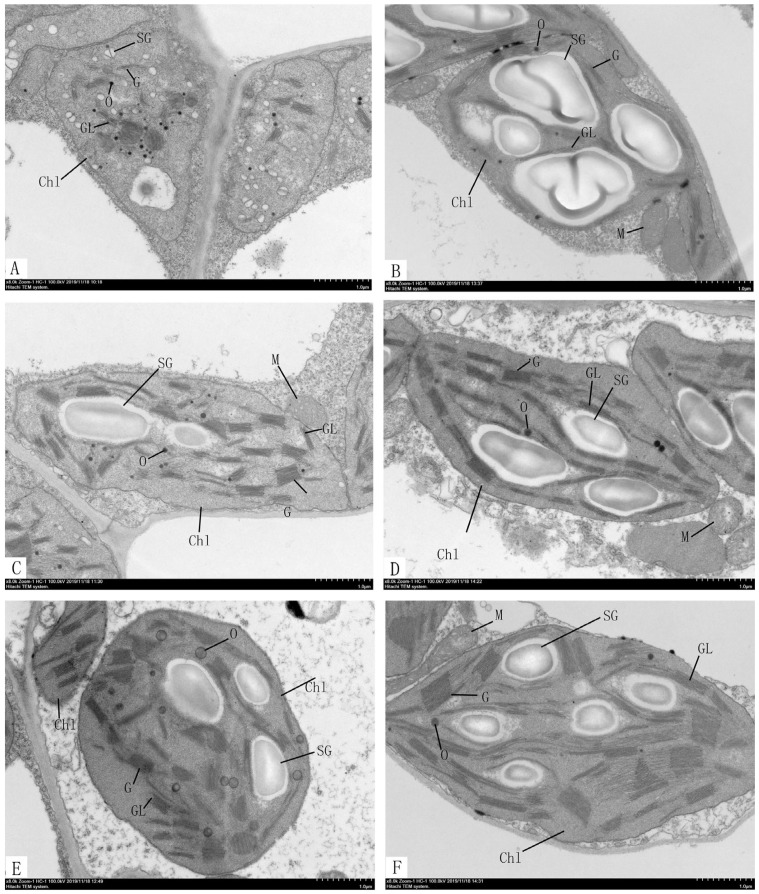
Observation of ultrastructure of chloroplast in the leaves of *ytg-1* and A18. Note: Chl: chloroplast, SG: starch granule, G: basal granule, GL: basal granule lamella, O: hungry granule, M: mitochondria. The magnification is 8000×. ((**A**,**B**): chloroplast at the yellow leaf stage. (**C**,**D**): green turningperiod. (**E**,**F**): late green period).

The chloroplasts in the leaves of *ytg-1* and A18 were observed and compared at the three stages (yellowing, green turning, and late green) using transmission electron microscopy. Compared with A18, *ytg-1* had fewer thylakoid grana lamellae in the chloroplast at the yellow leaf stage (Figure 5A,B), with irregular stacking, uneven grana shape, and disordered arrangement. In the green turning and late green period (Figure 5C–F), the number of grana lamellae of thylakoid increased, and the stacks were relatively neat; however, the grana were still in disorder. Starch granules existed in very small and numerous forms during the yellow leaf period, without the synthesis of large starch granules. In the green turning and late green periods, large starch granules were synthesized; however, the quantity was relatively small. Many hungry granules were observed (Figure 5A–F). The early yellowing and later turning green phenotype of *ytg-1* may be related to the early cleavage of chloroplasts and abnormal structure and order of grana lamellae of chloroplasts and thylakoid.

### 2.6. Genetic Analysis of the Phenotype of Leaf Color Variation in Mutant ytg-1

The leaves of F1 plants produced by reciprocal crossing between *ytg-1* and A18 were green in color (Table 2). The leaves of F1 and A18 backcross offspring were green. In total, 15 and 18 plants with yellow and green leaves were detected in the offspring of F1 backcrossing with *ytg-1*. χ^2^ testing revealed that the separation ratio of the latter corresponds to a Mendelian 1:1 separation ratio (χ^2^ = 1.2). Statistical analysis of the phenotype of the F2 population revealed that its segregation ratio fitted the expected Mendelian ratio of 3:1 (χ^2^ = 0.956). Based on the experimental results, we concluded that the yellow-to-green leaf trait of the snap bean mutant *ytg-1* is controlled by a single recessive nuclear gene, which was named *pvytg-1*.

### 2.7. Fine Mapping of pvytg-1

**Figure 6:**
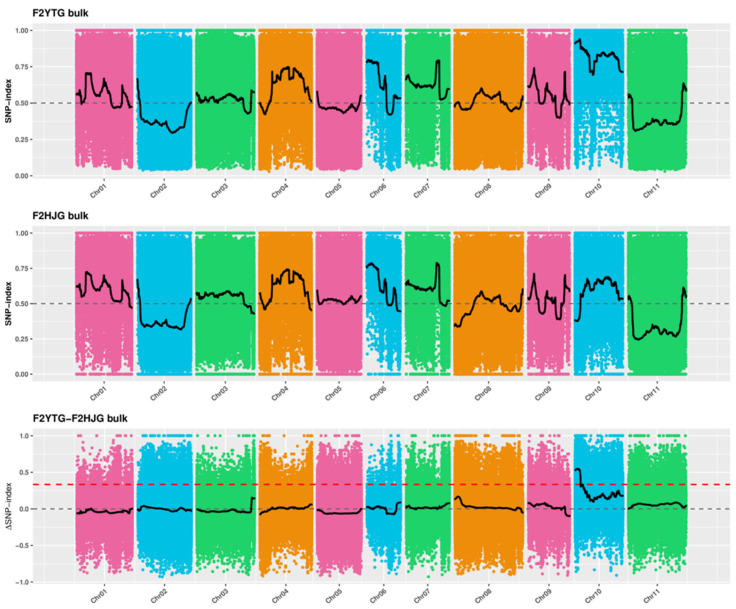
F2YTG bulk and F2HJG bulk SNP-index and correlation analysis diagram after fitting.

Using mutants *ytg-1* and HJG as parents, an F2 population was generated. From the F2 population, 50 plants each with green and yellow leaves were selected to construct respective DNA pools. DNA pools and parental DNA were sequenced. The sequencing results indicated that *pvytg-1* is located in the range of 0–5.5 Mb or 6.29–6.37 Mb on chromosome 10 (Figure 6).

**Figure 7:**
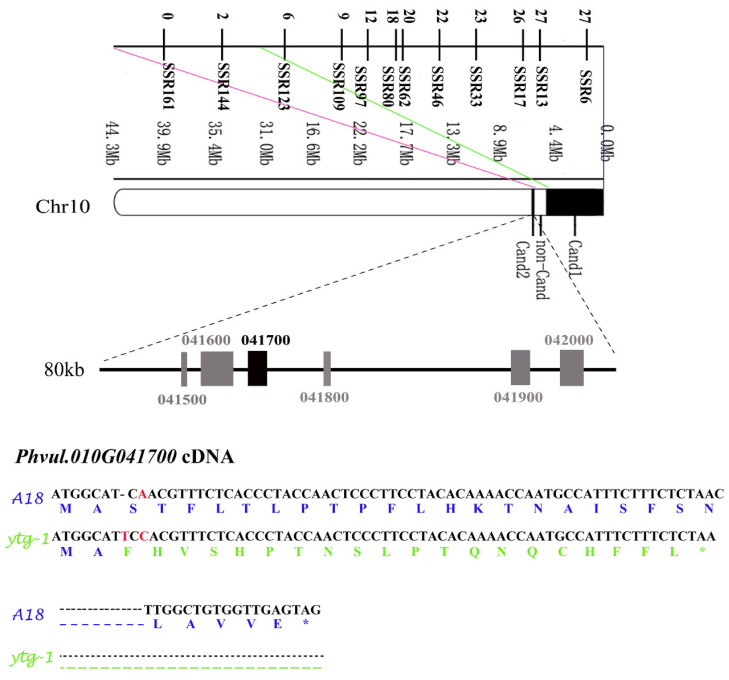
*pvytg-1* fine mapping interval and comparison of candidate gene cloning.

Based on the results of BSA-seq, SSR markers were developed at the intervals of 0–5.05 Mb (Cand1) and 6.29–6.37 Mb (Cand2). A total of 130 pairs of primers were uniformly designed on the Cand1 interval, and 10 pairs of SSR primers with stable and clear differential bands were screened on the parental DNA. A total of 33 pairs of primers were designed on the nontarget interval (non-Cand) of 5.05–6.29 Mb, and 2 pairs of SSR primers with stable and clear differential bands were screened. A total of 12 pairs of SSR markers were designed on the Cand2 interval; however, none of them exhibited polymorphism. These SSR primers were used to amplify the gene in 312 individual plants with a recessive phenotype in the F2 population. The relative positions of these markers were obtained using statistical analysis and recombination rate calculation. The results revealed that the number of recombinant plants with 10 SSR markers on Cand1 exhibited a decreasing trend, whereas the number of recombinant plants with two SSR markers on the nonCand interval was 2 and 0, indicating that these markers were located on the side of *pvytg-1* (Figure 7). It can be inferred that *pvytg-1* is located in the 80-Kb interval of Cand2.

### 2.8. Candidate Gene Screening and Sequence Analysis

Using the *P. vulgaris* v2.1 database (https://genome.jgi.doe.gov/portal/, accessed on 16 December 2024), the 6.29–6.37 Mb interval of fine mapping region located on chromosome 10 was analyzed, which contained a total of six genes. The length, location, and function of these six genes were annotated, including three functionally unknown genes and three functionally known genes (Table 3). *Phvul.010G041700* is a gene encoding chloroplast chaperone protein CPN10-1 involved in chloroplast protein folding, metabolic process regulation, and abiotic stress response and is related to chloroplast development. It is closely related to the morphological abnormalities of chloroplasts and changes in the number and structure of thylakoid reported in our study on chloroplast ultrastructure. Hence, it was speculated that *Phvul.010G041700* is a candidate gene for *pvytg-1*.

The coding sequence of *pvytg-1* was amplified and sequenced for *ytg-1* and A18. The CDS sequence of *Phvul.010G041700* exhibited a single base insertion (A7) in the *ytg-1* mutant, resulting in a frameshift mutation (Figure 7). The truncated protein has 23 amino acids. *Phvul.010G041700* has seven exons, and the insertion of single base occurs in the first exon. The *Phvul.010G041700* gene itself has a conserved protein domain of the cpn10 superfamily, located at the 57th to 132th amino acids. In the *ytg-1* mutant, due to the mutation of this gene, the gene sequence was prematurely terminated, with only the first 23 amino acids. Therefore, it does not have the conserved protein domain of the cpn10 superfamily, and hence does not have the function of this gene.

## 3. Discussion

Leaf color mutants are ideal materials for studying chlorophyll synthesis pathways, chloroplast structure and function, and photosynthesis mechanisms. They play important roles in related gene mapping, gene function research, and high light efficiency breeding [12]. In this study, ^60^Co-γ radiation was used to treat the seeds of A18; a mutant *ytg-1* that can be stably inherited was obtained through multiple generations of screening. By observing the plant, it was reported that, when the three-compound leaves grow out, they appear yellow. As the plant grows and develops, the leaf color gradually turns green. Previous researchers defined a type of mutant with decreased chlorophyll content in the early stages of leaf development and an almost normal level of chlorophyll content at the maturity stage as a yellow-to-green mutant [13].

In this experiment, *ytg-1* exhibited a decrease in chlorophyll content due to leaf color mutations, and its growth parameters were poorer than those of the wild-type. However, no significant difference was observed between the two plant types in terms of yield, and the number of pods per plant and 100-grain weigh were even higher than the wild-type. This was quite puzzling. Similar results have been reported in other studies, e.g., in leaf color mutants such as rice zebra leaves and rice yellow green leaves [14], which also exhibit higher grain length, width, and thousand grain weight than the wild-type. It was speculated that the reason for this may be due to the yellowing mutation affecting the synthesis of organic matter in plants and plant reproduction, which means that the nutritional growth of plants is limited and more energy is invested in reproductive growth. Therefore, the podding rate of snap bean increased, and more organic matter was used for seed formation. Overall, the yield of *ytg-1* was not significantly affected; therefore, this mutant has great potential as a leaf color marker for improved variety breeding.

By observing the ultrastructure of chloroplasts through transmission electron microscopy, it was speculated that the leaf color phenotype of *ytg-1* may be related to the lack of fixed shape, disordered arrangement, early cleavage of chloroplast, and low number and irregular stacking of thylakoid grana layers during the yellow leaf stage. Similar to the ultrastructure of most yellow leaf mutants, the morphology of chloroplasts and number and stacking pattern of thylakoid grana layers were altered. For example, in the study of chlorophyll-deficient mutants in rice, it was reported that the accumulation of thylakoid particles was irregular, and low chlorophyll content led to leaf chlorosis [6].

Chlorophyll is the main photosynthetic pigment. If chlorophyll synthesis is hindered, it may not accumulate normally, ultimately leading to changes in leaf color. Various leaf color types generally appear, among which yellowing is a more common phenotype. With the continuous advances in the research on the pathway of chlorophyll synthesis, researchers have divided the chlorophyll synthesis obstruction into two types. One is single site obstruction studied in snap bean and pineapple [15]. The other is multiple site obstruction reported in tobacco [16] and other plants. The mutant *ytg-1* leaves exhibited a gradual increase in chlorophyll content in the later stages. By measuring the content of intermediates of chlorophyll synthesis, it was reported that, during the yellow leaf period, the synthesis of Proto lX to Mg Proto lX in the synthesis pathway was hindered. This led to a decrease in the synthesis of Mg Proto lX to Chlide. However, during and after the green turning period, this hindered trend gradually decreased, and the leaves turned green.

The genetic patterns of different leaf color mutants vary, with most being recessive mutations controlled by nuclear genes. However, a small number of dominant and cytoplasmic gene mutations controlled by nuclear genes are observed. The genetic analysis in this study indicated that the yellow-to-green leaf trait of *ytg-1* is controlled by a single recessive nuclear gene. The BSA method was used to preliminarily identify the mutated genes, which can be located on the chromosomes and target intervals can be obtained. Currently, this is widely used in plants such as wheat [17,18] and pakchoi [19,20]. The mapping population was expanded, and the mutant gene was located in the 80-kb interval of chromosome 10. The analysis of six genes in the localization region indicated that the chloroplast chaperone protein CPN10-1 encoded by *Phvul.010G041700* was involved in chloroplast protein folding, metabolic process regulation, and abiotic stress response and was related to chloroplast development [5]. Previous studies have reported that, in *Arabidopsis*, chloroplast co-chaperone proteins also include CPN10, CPN20, and CPN60. Among them, CPN20 has been extensively studied. CPN60 in mitochondria has been extensively studied and is typically associated with the folding of ATP-binding protein [21,22,23]. Koumoto et al. assumed that CPN10-1 and CPN20 function independently [24]. CPN10-1 is expressed in the leaves and stems but not in the roots of *Arabidopsis*. It is also believed that CPN10-1 can accumulate in the dark, with leaves appearing yellow and quickly returning to green after exposure to light. In our study on mutant *ytg-1*, a simple incomplete shading device was used for the treatment. After 2 days, we reported that the shaded yellow leaves had begun to turn green, which is completely opposite to the response to light exposure in previous studies. It can be determined that the protein encoded by the yellow-to-green gene controlling the phenotype of *ytg-1* leaves is sensitive to light. It is speculated that the mutant gene *pvytg-1* causes misfolding of chloroplast-related proteins in the mutant, leading to changes in its function. The opposite perception mechanism appeared under light sensitivity, resulting in early leaf yellowing. This is consistent with the loss of function caused by the premature termination of candidate gene *Phvul.010G041700* mutations, which prevents normal response to light. The gradual turning green in the later stage is likely due to the presence of protein misfolding repair systems in the organism, which undergo misfolding repair as the leaves grow. This further indicates that protein misfolding caused by this mutation is reversible in the organism.

## 4. Materials and Methods

### 4.1. Materials

A18 is a high generation inbred line of snap beans. The yellow-to-green mutant of snap bean leaves ‘*ytg-1*’ is a mutated line and can be stably inherited. It was prepared by applying ^60^Co-γ radiation treatment to the dry seeds of A18, followed by open field planting, phenotype observation, and screening. ‘HJG’ is a high-level inbred line of snap beans with an appearance similar to that of A18, with pure green leaves. It was used for the hybridization with mutant ‘*ytg-1*’ to construct a fine-mapping population. All plant materials were planted in the horticultural experimental base of Heilongjiang University.

### 4.2. Determination of Agronomic Trait Parameters

When the plant grew to the first three-compound-leaf stage, 10 A18 and *ytg-1* plants were selected with similar growth status and hang tags. Starting from the 6th day, the plant height, main stem thickness, length of the first three compound leaves, and leaf width were measured every 3 days. At the maturity stage (60th day), yield characteristics including yield per plant, pod length, pod width, number of pods per plant, and 100-grain weight were measured. The yield and weight of fresh pods were measured using a 1% electronic balance. A Vernier scale was used to measure the length (back suture) and width of pods. Each measurement was repeated three times.

### 4.3. Determination of Photosynthetic Pigment Content

In total, five A18 and *ytg-1* plants with similar growth status were selected and labeled when they reached the three-compound-leaf stage. Starting from the 6th day, the three compound leaves were collected every 3 days as experimental materials. Overall, 0.5 g of compound leaves at different stages were extracted using 80% (*V/V*) acetone ethanol solution, and the absorbance was measured at 470, 645, and 663 nm using a UV spectrophotometer. Each measurement was repeated three times. The contents of total chlorophyll, chlorophyll a (Chl a), chlorophyll b (Chl b), and carotenoids were evaluated as described previously [25].

### 4.4. Determination of the Intermediate Metabolites in Chlorophyll Biosynthesis

The sampling process was the same as described in the previous section for the determination of photosynthetic pigment content. The ALA content was determined as described by [26]. The PBG, UrogenIII, and CoprogenIII contents were determined using the method by [27]. The ProtoIX, Mg-protoIX, Pchlide, and Chlide contents were determined as described by [28]. The experiment involved three biological replicates.

### 4.5. Observation of the Ultrastructure of Chloroplasts

Five A18 and *ytg-1* plants with similar growth status were selected. When the plant grew to the first three-compound-leaf stage, the tag was hung. The compound leaf samples were collected on the 6th (yellow leaf period), 15th (green turning period), and 21st (late green period) days as experimental materials for transmission electron microscopy.

### 4.6. Genetic Analysis of Leaf Yellow-to-Green Mutant Characteristics

A18 and *ytg-1* were used as parents to conduct a six-generation cross. The phenotypic characteristics of F1, BC1, and F2 generations were investigated, and the segregation ratio was determined using χ^2^ (Chi square) test.

### 4.7. Bulked-Segregant Analysis Coupled with Whole-Genome Sequencing (BSA-Seq) Analysis

*ytg-1* and HJG were hybridized to construct an F2 generation mapping population. In total, 50 plants with green leaf phenotypes in the F2 population were selected; DNA was extracted from the leaves and mixed to construct a mixed pool, denoted as F2HJG. Overall, 50 plants with yellow leaf phenotypes in the F2 population were selected; DNA was extracted from the leaves and mixed to construct a mixed pool, denoted as F2YTG. DNA from the leaves of *ytg-1* and HJG was extracted and labeled as YTG and HJG, respectively.

The construction and inspection of the library, as well as the sequencing work on the computer, were completed by Hangzhou Lianchuan Biotechnology Co., Ltd. (Hangzhou, China). Quality control of offline data, comparison of reference genomes, and detection and annotation of SNP and InDel were performed. Next, the SNP index was calculated, and the difference in the offspring mixed pool was observed. The regions with significant differences in SNP index between the two offspring mixed pools were selected, and the target trait region was located on the chromosome of the species (i.e., the segments on the chromosome were determined). Finally, candidate genes were selected within the segment and functionally annotated.

### 4.8. Fine Mapping of pvytg-1 Mutated Gene

In total, 312 recessive individuals from the F2 generation population were selected for fine mapping. Based on the preliminary candidate regions identified using BSA-seq, SSR markers were designed for localization interval validation analysis. The primer design is evenly distributed within the candidate interval, and the base sequence was downloaded from the *Phaseolus vulgaris* v2.1 (common bean) database (https://phytozome-next.jgi.doe.gov/, accessed on 16 December 2024). The primers [Primer Premier 6.0 (Premier Biosoft International, Palo Alto, CA, USA)] were designed and sent to Tianjin Jinweizhi Biotechnology Co., Ltd. (Tianjin, China) for synthesis (Table 4). The primers were used for polymorphism screening between two parents and two populations. Markers with polymorphism in both parents could be used to further narrow down the initial localization interval.

MAPCHART software (version 3.0) (Wageningen, The Netherlands) was used to analyze the linkage relationship between SSR markers and *pvytg-1*. Finally, TBtools software (version 4.1) (Nanjing, China) was used to construct a genetic linkage map from polymorphic SSR markers and segregation data linked to genes.

### 4.9. Gene Cloning and Sequence Comparison

Total RNA was extracted from the leaves using a plant whole RNA extraction kit (Tiangen, Beijing, China). cDNA was synthesized using a Reverse transcription kit HiScript^®^ II Q Select RT SuperMix for qPCR (+gDNA wiper), (Novozan, Nanjing, China). Based on the selected candidate gene, corresponding primers were designed based on their full-length CDS sequence, and the cDNA sequences of mutant *ytg-1* and wild-type A18 were used as templates for PCR amplification. The PCR primers used are shown in Table 5. PCR products were sent to Tsingke Biotechnology Co., Ltd. (Beijing, China) for sequencing. After checking and splicing the sequencing results using DNAMAN software (version 4.0) (LynnonBiosoft, San Ramon, CA, USA), heterotopic point alignment analysis for the difference in sequence was performed to identify mutation sites and determine the type of mutation.

## 5. Conclusions

In this study, a yellow-to-green mutation of snap bean leaf color was created. The mutant trait belongs to the stage deficient restoration type of total chlorophyll, and the decrease in chlorophyll during the yellow leaf stage is caused by the inhibition of Proto IX to Mg Proto IX synthesis. However, this leaf color mutation trait has little impact on plant yield and can be applied as a marker trait in hybrid breeding. Study on the mutated gene revealed that the yellow-to-green mutation of the leaf color is controlled by a single dominant nuclear gene, and it is located in the 80-kb interval of chromosome 10. Among them, *Phvul.010G041700*, which encodes chloroplast chaperone protein CPN10-1, loses translation function due to the insertion of a single base in the coding sequence and can be used as a candidate gene. This study can lay the foundation for marker trait breeding.

## Figures and Tables

**Table 1:** Comparison of yield traits between mutant *ytg-1* and A18.

Strain	Pod Length (cm)	Pod Width (cm)	Single Plant Yield (g)	Number of Pods Per Plant (number)	100 Grain Weight (g)
*ytg-1*	14.87 ± 0.06 **	1.01 ± 0.04	166.67 ± 5.77	37.00 ± 2.65 *	38.38 ± 0.22 **
A18	16.77 ± 0.15	1.05 ± 0.01	150.00 ± 10.00	31.33 ± 1.15	37.11 ± 0.11

Note: In the same column, *: *p* < 0.05, **: *p* < 0.01.

**Table 2:** Genetic analysis of the yellow-to-green leaf phenotype.

Generations	Total	Green Leaves	Yellow to Green Leaves	Segregation Ration	χ^2^
P1 (*ytg-1*)	50	0	50		
P2 (A18)	50	50	0		
F1 (P1 × P2)	32	32	0		
F1 (P2 × P1)	28	28	0		
BC1 (F1 × P1)	33	18	15	1.2:1	1.2
BC2 (F1 × P2)	29	29	0		
F2	1233	916	317	2.89:1	0.956

**Table 3:** Analysis of candidate genes in Cand2 region on chromosome 10.

Gene	Name	Location	Function Notes
Phvul.010G041500	-	6303589–6304203	-
Phvul.010G041600	-	6307176–6312920	-
Phvul.010G041700	CPN10-1	6314835–6318150	10 kDa chaperonin 1, chloroplastic
Phvul.010G041800	ATL4	6329479–6330555	E3 ubiquitin-protein ligase ATL4
Phvul.010G041900	-	6358186–6361597	-
Phvul.010G042000	At5g59540	6365314–6369368	1-aminocyclopropane-1-carboxylate oxidase homolog 12

**Table 4:** Marker primer sequences closely linked to the target gene.

Markers	Upstream Primer (5′-3′)	Downstream Primer (5′-3′)	Location
SSR6	GCAGTTGCAGTCATTGTATAG	GGAAAGAAATTGGGAAAGACAG	433,325–433,425
SSR13	CCTCATTGACTTGCTTCATT	CAACTCCTTGTTTGACCAA	1,113,712–1,113,812
SSR17	CTGCTTACATCTTTGTCCTTC	CTAGTGTTGACCATTTGAGTG	1,364,003–1,365,003
SSR33	TAGTTGGTGGTCGTCCTAT	TGGTCATTCTTAGTGGTTGT	1,950,571–1,950,871
SSR46	GATGGAATGTCAGTGAGGTA	CAGTGTTGTAGAGGTTGGA	2,402,344–2,402,644
SSR62	TTGGAACACAACCACTCAT	ACTCCTTTAGAAACCTCTCTC	2,833,661–2,833,961
SSR80	CTCTCCACTCTTCTTCTTCTT	GAGGAACTGCGATAACTAATG	2,912,191–2,912,318
SSR97	CCTACCCATTCTTGAATAACC	CTTGCTCAGCTCACTCTC	3,054,694–30,554,714
SSR109	TCCACTGCTACTGTGTCTA	CTCCAATCCATCATCATCATC	3,497,326–3,497,626
SSR123	ACATCGTGACGGAGAACA	AGTAGGAAGCAATGCCATC	4,571,601–4,571,619
SSR144	TCTTCTTCCTCTCACTTCTC	CTCCGCATAAGCAGACAA	5,315,083–5,315,102
SSR161	CACGATGGTTGGAGTTAGAA	AGTGGCTATCTATATGTGGAAGG	6,092,486–6,092,505

**Table 5:** Primers for *pvytg-1* cloning.

Gene ID	F-Primer (5′-3′)	R-Primer (5′-3′)
*pvytg-1*	AGCAACTGAGTAGATAAACCTAA	AGTTGGTCTTATTAACACCATTT

## Data Availability

The original contributions presented in this study are included in the article. Further inquiries can be directed to the corresponding authors.
